# A century of morphological variation in Cyprinidae fishes

**DOI:** 10.1186/s12898-016-0104-x

**Published:** 2016-10-20

**Authors:** Stephen J. Jacquemin, Mark Pyron

**Affiliations:** 1 Department of Biological Sciences, Dwyer Hall, Wright State University-Lake Campus, Celina, OH 45822 USA; 2 Department of Biology, Aquatic Biology and Fisheries Center, Ball State University, Muncie, IN 47306 USA

**Keywords:** Geometric morphometrics, North American fish morphology, Long term morphology, Global change, Long term hydrological variation, Habitat alteration, Contemporary evolution

## Abstract

**Background:**

Aquatic habitats have been altered over the past century due to a variety of anthropogenic influences. Ecomorphology is an area of aquatic ecology that can both directly and indirectly assess the effects of habitat alterations on organisms. However, few studies have explored long term trends in morphological variation. Long term changes in morphology can potentially impact niche and ultimately contribute to organismal success and the ecosystem. Therefore, in this study we assessed long term morphological variation with body size, sex, time, and hydrology using museum collections of five species of Cyprinidae (Minnows) from lentic and lotic systems over the past 100 years to gain insight into long term patterns in morphology.

**Results:**

Variation in Cyprinidae morphology tended to relate to: body size—indicating strong allometric growth patterns with robustness of larger individuals; sex—indicating a level of fecundity selection for deeper bodies in females compared with males; and year—indirectly suggesting responses to habitat changes over the past century. In lotic ecosystems, Cyprinidae morphology tended to be more fusiform in conjunction with lower mean annual discharge or higher variation in discharge. In lentic ecosystems, change in morphology was observed but no historic habitat variables were available to discern potential mechanisms. Interestingly, not all species responded in the same magnitude or directionality.

**Conclusions:**

Long term changes in morphological variation provide a link to exploring functional relationships between taxa and their environment and have implications for understanding ecosystem attributes, community assembly patterns, and conservation.

## Background

Freshwater ecosystems have become increasingly altered over the past several centuries through a variety of anthropogenic changes to watersheds that have resulted in degraded physical habitats, declines in water quality, and disruption of flow regimes on both local and regional scales [1–3]. Understanding the potential effects of widespread ecosystem alterations on biota and their implications have become primary conservation issues. Central to understanding the impact of changes to habitats over the past century are species-environment relationships [4], particularly how organismal morphology may play a part in the functional role of an individual in an ecosystem. These larger scale relationships between morphology and environment can improve predictability and understanding of potential assemblage and ecosystem level attributes [5–7]. However, few studies have used long term collections or datasets to assess what variables may relate to variation in morphology over a long term period. Therefore, the purpose of this study was to apply an exploratory technique incorporating a suite of tests to identify potential morphological covariates in fishes over the past century.

Freshwater fishes display a range of morphological adaptations across a wide variety of physiological states and environmental conditions as a result of genetic divergence and/or phenotypic plasticity [5, 8, 9]. Physiological influences are frequently identified as morphological relationships with body size (allometry), diet (feeding performance), and sex (sexual selection) while plasticity tends to respond to local environmental variation related to niche patterns of resource utilization, behavior, and/or habitat use [5, 8–11]. Typically, as fishes move through their ontogeny there are allometric trends in shape [12, 13]. These allometric trajectories can follow different patterns in individual species but are often shown to relate to body depth and robustness of an individual [12, 14]. These changes with ontogeny can be further explored in light of sexual dimorphism as individuals reach sexual maturity, whereby females tend to take on a deeper body shape compared with males. In addition to body size and sex, diet and resource utilization affect morphology, whereby particular dietary items can induce morphological change within or among populations [8]. Lastly, all of these factors can be complicated by environmental variation with phenotypic plasticity. For example, Langerhans et al. [15] identified increasingly fusiform body shapes of Neotropical fishes in main channel habitats compared to lower flow lagoon habitats coinciding with variation in flow conditions. Similarly, Haas et al. [16] found that deeper-bodied *Cyprinella* (Cyprinidae) are indicative of reservoir compared to riverine habitats. More recently, Dugas et al. [17] studied body streamlining (fusiform) with river velocity in *Cyprinella whipplei* males and did not find positive correlations but did suggest that deeper body shape may provide increased maneuverability during male–male interactions during combat and that this may be of greater importance than increased streamlining. Regardless, while these field documented effects of local habitats influence on morphology are varied they have been reproduced and manipulated in laboratory experiments [18]. Yet, while these documented trends in physiology and plasticity exist in contemporary studies, it is impossible to quantify everything that drives body shape trends. Further challenging is dealing with questions of long term organismal changes [19] where a paucity of biological specimens and/or environmental information impedes long term morphological studies.

Natural history museum collections may provide the best opportunity to observe and test for some of these long term changes in morphological traits of taxa concurrent with habitat variation. Biological and environmental data are most readily available in the form of historical museum collections of preserved fish specimens (potentially from 1880 to present [20, 21]) and associated specimen lot description tags. For example, a recent study utilizing museum collection lots and data identified rapid change in the morphology of a stream fish in the decades that followed construction of a series of reservoirs in the United States [19]. And while these long term collections that are available do not encompass all taxa from all locales, these resources can help facilitate ecological hypotheses across a large temporal scale of taxa, assemblages, and ecosystems [22].

Specific to long term morphology of fishes, morphological variation that is correlated with physiological parameters such as body size or sex can be directly assessed from measurement or dissection of preserved specimens and analyzed by collection year. These types of studies become more accessible compared with those invoking plasticity or immigration trends with environmental change as local habitat conditions at the time of these long term collections may not be as readily apparent with variation in detailed collection records. One interesting solution to sparse habitat records is to use time as an indirect proxy for habitat change and follow up with historical climate change data of precipitation, temperature, landscape development trends, or with direct records such as flow regime (daily hydrology data available for many streams from 1900 to present—http://waterdata.usgs.gov/).

Global climate change models were particularly helpful in facilitating our understanding of ecosystem responses to changes in precipitation, temperature, and human landscape impacts [23]. Of particular interest are changes in flow regime, as flow regime provides an overarching influence on aquatic ecosystems [1], either directly in streams or indirectly for lakes and can serve as a proxy for habitat [24]. The effects of flow regime changes on freshwater fishes have been documented in numerous taxa and communities (reviewed by Poff and Zimmerman [25]) and provide inferences into population responses to environmental alterations [16, 26]. Alterations to flow regimes have been linked to physical habitat homogenization and degradation [27], changes in aquatic assemblages [28], reductions in ecological community resilience [29], changes in assemblage functional attributes [30, 31], changes to structure of assemblage level morphological diversity [32], and long term hydrology effects on contemporary assemblages [33]. However, fewer studies have linked long term changes in freshwater fish taxa with variation in environment [34].

Despite numerous studies that have established relationships between morphology and suites of abiotic and biotic factors, the patterns that emerge from long term tests for these relationships are virtually unknown. Therefore, the objective of this study was to describe shape variation for five species in the family Cyprinidae (Minnows) relative to body size, sex, time, and environment (e.g. hydrology where available) over 100 years using long term collections from both lotic and lentic sites. We predicted that long term collections would exhibit morphological signal as a result of both physiological and environmental influences. Ultimately, ecomorphological perspectives may improve our understanding of shape variability while providing potential links with functional performance in changing ecosystems.

## Methods

### Fish and morphology

**Fig. 1:**
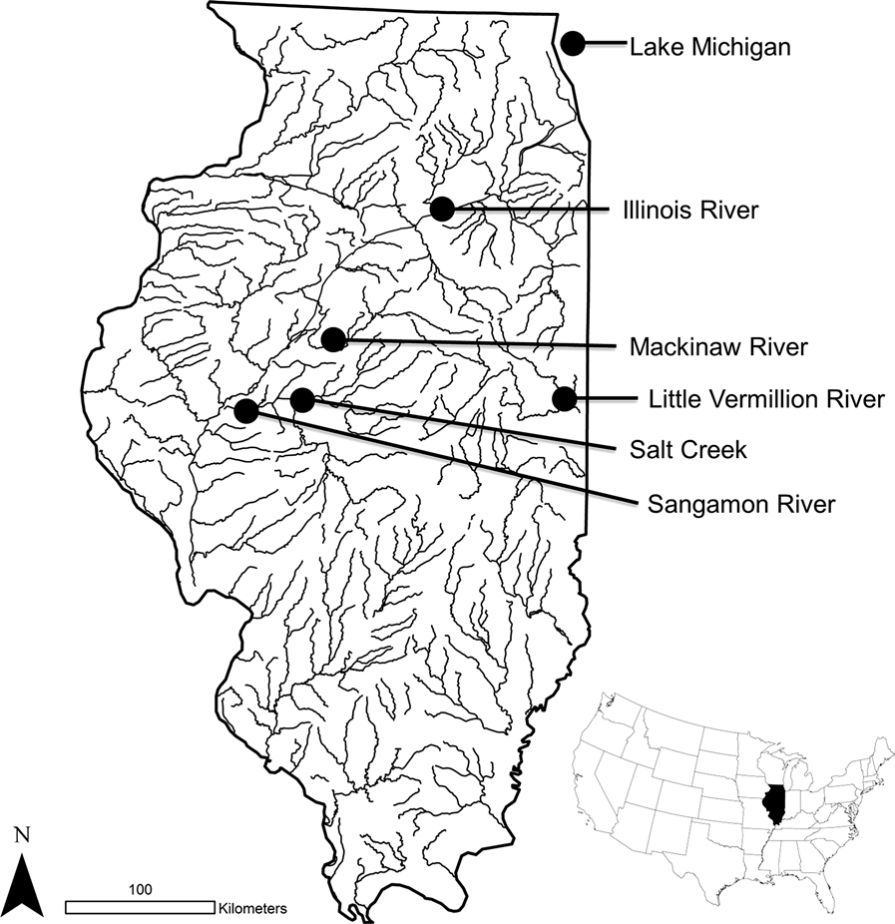
Map of collection localities and USGS stream gauging stations

**Fig. 2:**
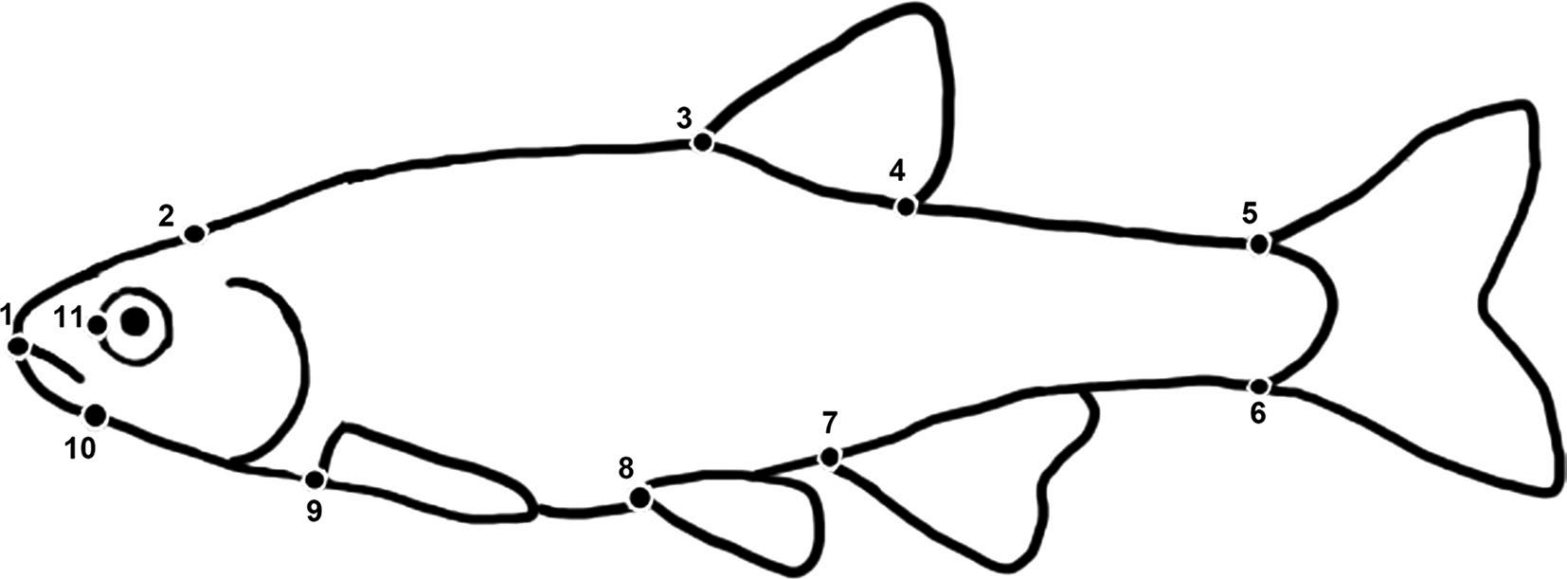
Location of morphology landmarks. Landmarks correspond to anterior snout (*1*), superior margin of head (*2*), anterior dorsal origin (*3*), posterior dorsal origin (*4*), superior posterior caudal peduncle (*5*), inferior posterior caudal peduncle (*6*), anterior anal fin origin (*7*), anterior pelvic fin origin (*8*), superior pectoral fin origin (*9*), ventral opercular isthmus (*10*), and anterior medial edge of orbital socket (*11*)

Museum collections of five species of Cyprinidae from the Illinois Natural History Survey (INHS) were selected based on long term repeated collections of approximately ten individuals representing both males and females from each collection effort. Species found to meet this criteria were Striped Shiner (*Luxilus chrysocephalus*), Redfin Shiner (*Lythrurus umbratilis*), Emerald Shiner (*Notropis atherinoides*), Sand Shiner (*Notropis stramineus*), and Bluntnose Minnow (*Pimephales notatus*) (see Appendix for INHS catalog numbers). Collection dates ranged from 1899 to 2006. All collections were in Illinois, USA (Fig. 1; Table 2) and included long term biological monitoring sites on the Sangamon River (Champaign/McLean Co.), Mackinaw River (Tazewell/Woodford Co.), Little Vermillion River (Vermillion Co.), Salt Creek (Logan/DeWitt Co.), Illinois River (LaSalle/Grundy Co.), and Lake Michigan (Cook Co.). Individuals were photographed using a tripod-mounted digital Nikon D70 camera (Nikkor AF-S DX Macro Zoom Lens) against a grid display for scale reference. Images were digitized by one person (SJJ) using a series of 11 predefined landmarks [14] (see Fig. 2 for landmark placement and description) within the software tpsDig [35]. To account for bent specimens in collection jars, four additional lateral landmarks, not included in the shape analyses, were added along the midline of each individual to digitally unbend specimens using the ‘unbend function’ within tpsUtil [36]. Only adult individuals were photographed and digitized. Centroid size was calculated to infer body size of digitized individuals [37]. Sex was determined by gonad inspection. Only collections preserved for a minimum of five years were used to avoid preservation bias in shape that can occur during initial formalin fixation and long term ethanol storage using standard museum preservation protocols [38].

### Habitat of rivers and lakes

To characterize flow habitat of lotic sites, daily discharge data from United States Geological Survey (USGS) stream gauging stations (http://waterdata.usgs.gov/) were used to describe site flow conditions (Table 1). Gauging stations were selected from the same streams (less than 15 km from site) as biological collections. Daily discharge data were analyzed using two approaches. First, annual mean and variation (coefficient of variation) were calculated from daily discharge data spanning a full calendar year prior to collection to specifically capture the flow conditions leading up to each individual collection effort. Second, overarching site level trends using all available years (including both collection and non-collection years) were analyzed for larger scale emergent patterns with year using the Indicators of Hydrologic Alteration (IHA) [39] flow regime methodology (calculated using IHA software ver. 7.1, The Nature Conservancy). For this broad scale hydrology analysis, all available years were used to provide a hydrology description of the entire site depicting the dominant hydrological trends through time that each lotic fish population had experienced over time. IHA calculates 32 hydrological variables in five categories including magnitude, frequency of occurrence, duration of flow condition, timing, and flashiness [1] and provides a measure of change in these individual variables with time using regression analysis. These IHA variables were identified as ecologically relevant, particularly with regard to feeding, reproduction, maturity, timing of life cycle periods, and stress [39]. In summary, magnitude is a measure of wet volume and can serve as an indication of high versus low flows. Frequency of occurrence is a measure of how episodic extremes in flow magnitude (high or low) occur. Duration of flow condition is a measure of the longevity of different flow events. Timing is a measure of when in a Julian water year extreme conditions occur. The flashiness category is a measure of the rate of change attributed with variation in flow conditions. Departures from the natural flow regime were linked to anthropogenic modifications of rivers, either directly or indirectly [1, 39]. Since hydrological information was only available for lotic sites, and no direct habitat information was available for lentic sites, only time was able to be used as an indirect approximation of habitat change [40]. Time also served as a covariate in lotic system analyses. Although not mechanistic in nature, time is indirectly linked to changes in water quality and habitat in the Midwestern United States over the past century [40].

**Table 1:** List of USGS gauging stations by drainage basin and include years

Site code	Drainage basin	Site	Hydrology years
USGS 05582000	Illinois River	Salt Creek at Greenview	1940–2003
USGS 05583000	Illinois River	Sangamon River at Oakford	1910–2005
USGS 05543500	Illinois River	Illinois River at Marseilles	1919–2003
USGS 05568000	Illinois River	Mackinaw River at Green Valley	1921–2000
USGS 03339000	Wabash River	Little Vermillion River at Danville	1914–2002

### Modeling morphology

General procrustes analysis (GPA; least squares method) was used to superimpose digitized individuals to a reference or consensus shape by removing effects of scaling, rotation, and translation, allowing comparisons among individuals [36]. Relative Warp Analysis (RWA) was performed in tpsRelw [37] and used to assess total morphological variation among individuals. RWA is essentially a morphometric principal components analysis of relative landmark positions (using variance/covariance matrix) and produces a series of orthogonal axes ranked by the amount of shape variation explained [41]. All shape axes were retained for analyses in this study. Two sets of RWA analyses were performed on individuals, including a family level analysis which incorporated all individuals from all species to test for macro level interspecific effects across species as well as a series of separate RWAs for each species at each site to test for intraspecific variation. Shape axes were treated as dependent variables in multivariate analysis of covariance (MANCOVA) models to test for relative influence of the following factors: species, site, body size, hydrology, sex, year, and all biologically relevant interactions (interpreted through heterogeneous slopes; [42]). Given the exploratory nature of the study, the following terms and interactions were included in the family combined model: species, site, body size, sex, year, daily discharge mean, daily discharge variation, body size * species, sex * species, year * species, daily discharge mean * species, and daily discharge variation * species while the following terms and interactions were included in the species specific models: body size, sex, year, daily discharge mean, daily discharge variation, body size * sex, body size * daily discharge mean, body size * daily discharge variation, daily discharge mean * year, daily discharge variation * year, daily discharge mean * sex, daily discharge variation * sex, and sex * year. Initial models revealing insignificant interaction terms were rerun without interaction terms. An unbalanced MANCOVA design was used to accommodate some limited cases of missing data of certain columns, such as where hydrological records were missing [43]. We felt inclusion of all samples and time periods were relevant as they contained information particular to specific trends (e.g. testing for temporal trends in cases where hydrological information was missing). Prior to a given terms inclusion in the family or species level MANCOVA models, issues with multicollinearity were assessed using variance inflation factor (VIF) statistics with a cutoff of 2.5 [44]. In instances where a variable exceeded this VIF threshold, that variable was dropped from the model and the model was rerun. Wilk’s lambda was used to calculate *F* values and Pillai’s partial eta^2^ was used to infer effect size by variance explained. Partial eta^2^ reflects the proportion of variation explained by a given independent variable while parsing out variation from other independent variables. As such, partial eta^2^ values are not necessarily able to be summed to 1.0 but can provide a degree of variation explained, particularly relative to other variables included in the model [45]. In addition to relative warp analyses with MANCOVA models, total morphological disparity was calculated to compare species. Disparity indices can be useful in discerning relative degrees of variability between species or groups and may contribute to our understanding of specific variation over long sampling time periods or provide general evolutionary information to larger order taxonomic groups [46, 47]. Disparity was calculated from Procrustes coordinates using a randomized residual permutation procedure using 1000 permutations [48]. All models were implemented in the statistical language R (version 2.14.1, R Development Core Team, 2011) using the heplots [45], fmsb [43], geomorph [48], and base stats packages. Thin plate spline deformation grids [49] (produced in tpsRegr [50]) were used to visualize shape variation indicated in MANCOVA models. Alpha was 0.05 for all tests of significance.

## Results

Museum collections of Cyprinidae were from long term (100+ year) collections at five lotic sites (Sangamon River, Salt Creek, Little Vermillion River, Mackinaw River, and Illinois River), all with adjacent USGS daily discharge monitoring sites, and one lentic site (Lake Michigan). Specimen collection dates ranged from 1899 to 2006 and spanned 98 to 106 collection years (mean number of collection events per site = 8.5) across the sites (Table 2; Appendix). Number of individuals per collection event included in the analyses ranged from 9 to 34 (mean number of individuals = 13.9). All sites included the available range of body sizes of sexually mature individuals of both males and females. United States Geological Survey (USGS) hydrological archives of daily discharge ranged from 63 to 96 consecutive years at the five lotic sites, with the earliest data collected in 1910 (Table 1). Indicators of Hydrologic Alteration analyses revealed significant alterations for all sites across the five primary IHA categories, including frequency and duration of pulses, magnitude and duration of annual extremes, magnitude of monthly conditions, rate and frequency of water condition changes, and timing of annual extreme conditions (Appendix). Mean number of hydrological alterations (of 32 possible) at sites was 11.4 (range of 1–23 alteration types) with varying degrees of magnitude, indicating that sites were variable in numbers of alterations and the extent these alterations modified the flow regime (Appendix). Overall, Cyprinidae morphology was related to a combination of variables and interaction terms, including body size, sex, hydrology (lotic sites), and time but was not uniformly consistent across species (Tables 3, 4).

**Table 2:** List of taxa with collection site, collection year, and number of collections per site

Species	Collection site	Collection years	Number of collections	Mean sample (±SE)
Striped Shiner *Luxilus chrysocephalus*	Sangamon River	1901–2001	9	n = 15 (2.4)
Redfin Shiner *Lythrurus umbratilis*	Little Vermillion River	1899–2002	9	n = 14 (2.1)
Emerald Shiner *Notropis atherinoides*	Illinois River	1897–2003	8	n = 13 (1.2)
	Lake Michigan	1900–1999	7	n = 15 (3.6)
Sand Shiner *Notropis stramineus*	Sangamon River	1901–2005	9	n = 17 (3.4)
	Mackinaw River	1901–2000	11	n = 14 (2.4)
Bluntnose Minnow *Pimephales notatus*	Salt Creek	1900–2003	9	n = 10 (1.0)
	Lake Michigan	1900–1998	6	n = 13 (4.5)

**Table 3:** Results from family level MANCOVA model assessing relationships between morphological variables and body size, hydrology, sex, year, and interactions for five species

Family	Effect	Variance explained	*F*	d.f.	*P*
Cyprinidae	Species	0.70	2.8	72, 2004	<0.001
	Body size	0.45	22.7	18, 498	<0.001
	Sex	0.11	3.6	18, 498	<0.001
	Year	0.08	2.4	18, 498	<0.001
	Discharge mean	0.11	3.2	18, 498	<0.001
	Discharge CV	0.08	2.3	18, 498	<0.001
	Body size * species	0.10	2.8	72, 2004	<0.001
	Sex * species	0.06	1.7	72, 2004	<0.001
	Year * species	0.11	3.4	72, 2004	<0.001
	Discharge mean * species	0.08	2.5	72, 2004	<0.001
	Discharge CV * species	0.09	2.6	72, 2004	<0.001

**Fig. 3:**
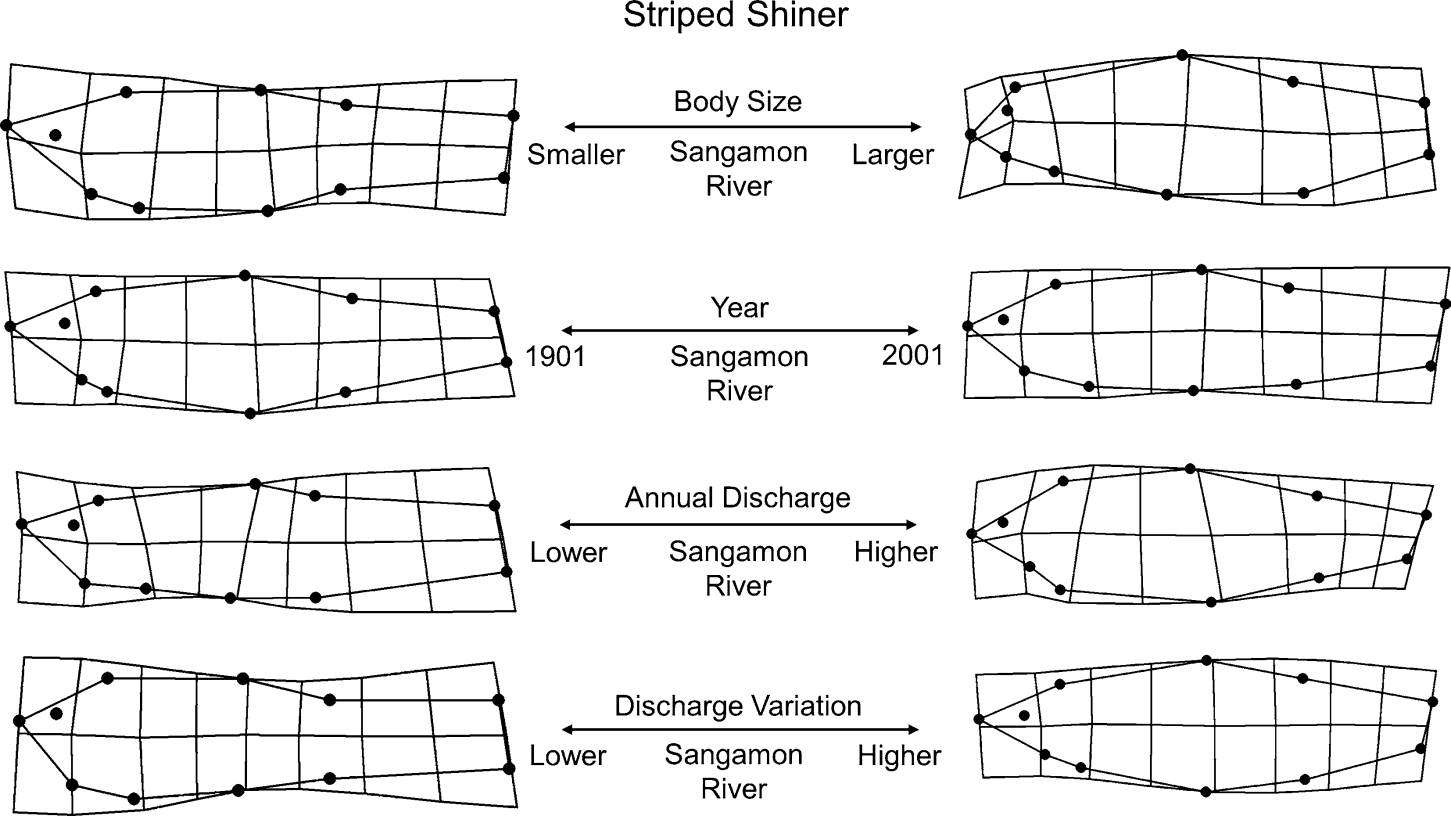
Striped Shiner morphological variation along significant predictor gradients using thin plate spline deformation grid extremes (magnified ×3 for visualization)

**Fig. 4:**
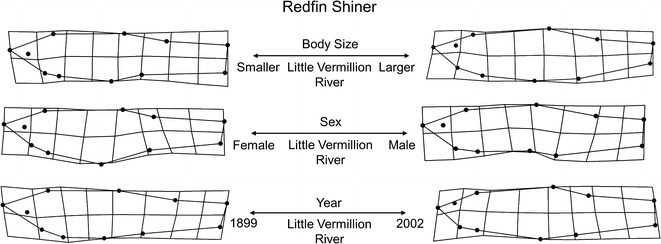
Redfin Shiner morphological variation along significant predictor gradients using thin plate spline deformation grid extremes (magnified ×3 for visualization)

**Fig. 5:**
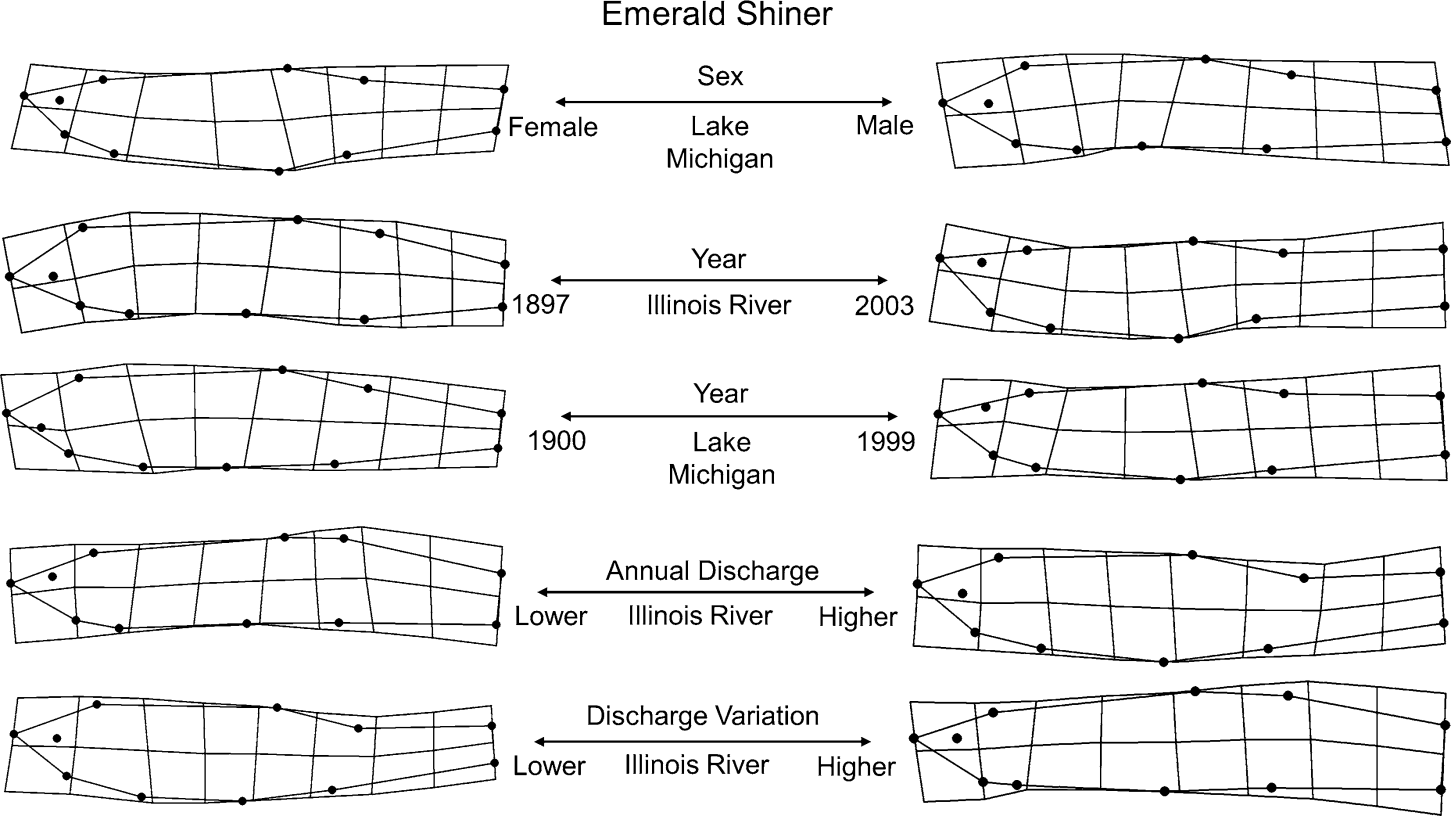
Emerald Shiner morphological variation along significant predictor gradients using thin plate spline deformation grid extremes (magnified ×3 for visualization)

**Fig. 6:**
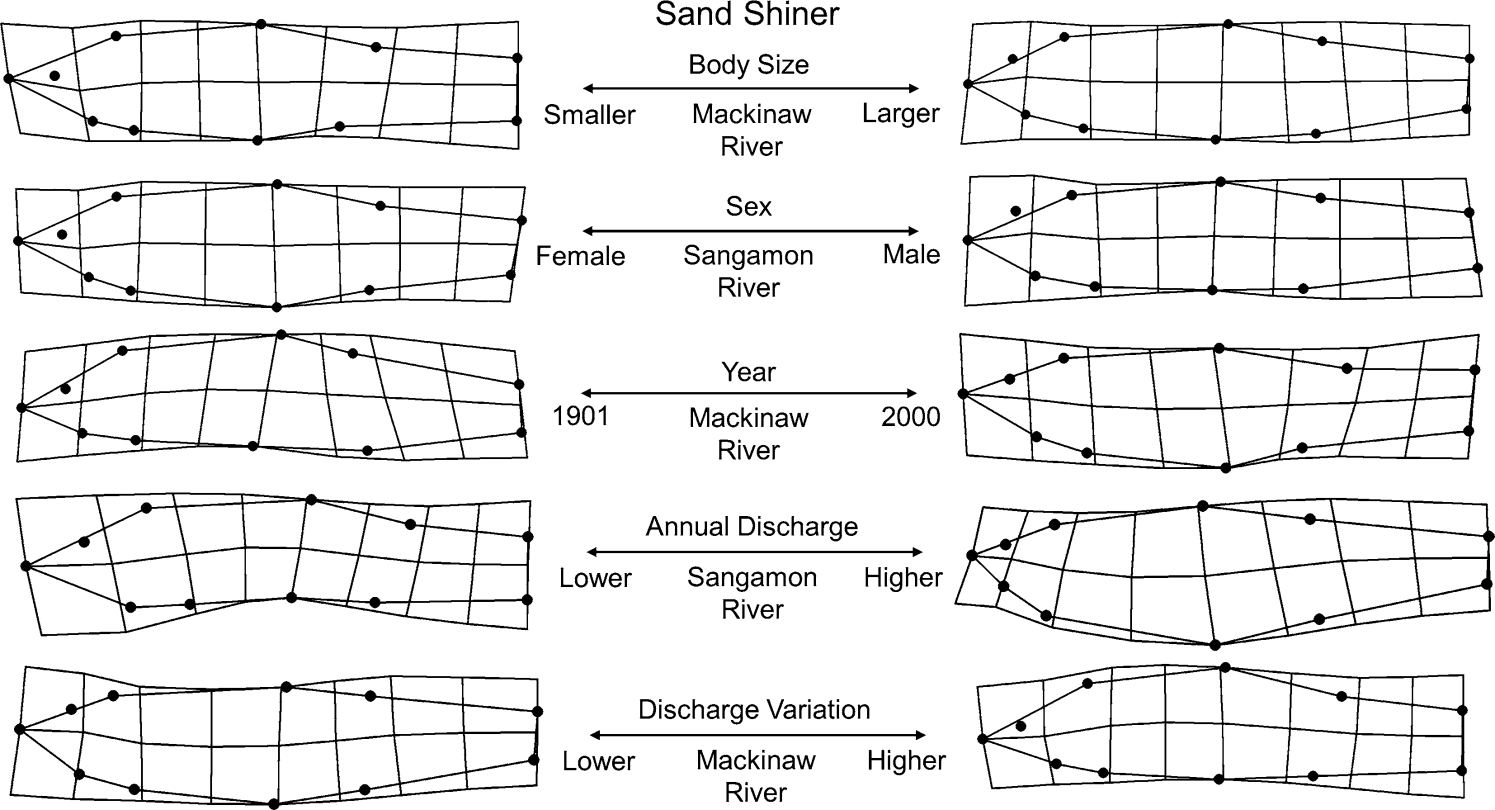
Sand Shiner morphological variation along significant predictor gradients using thin plate spline deformation grid extremes (magnified ×3 for visualization)

**Fig. 7:**
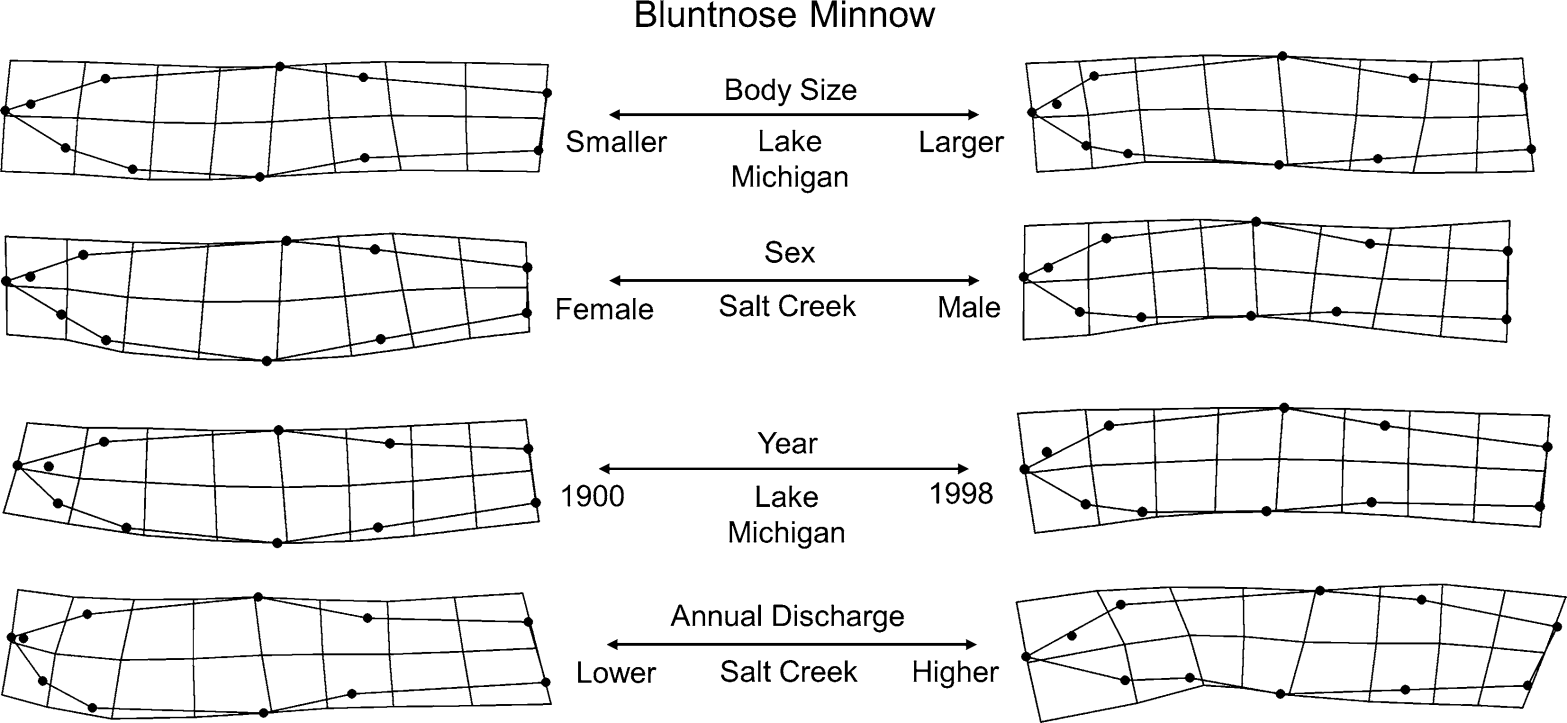
Bluntnose Minnow morphological variation along significant predictor gradients using thin plate spline deformation grid extremes (magnified ×3 for visualization)

See Table 4 for taxon specific analyses and Figs. 3, 4, 5, 6, 7 for visualizations of shape changes in individual species

**Table 4:** Results from MANCOVA models assessing relationships between morphological variables and body size, hydrology, sex, year, and interactions for five species

Species	Site	Effect	Variance explained	*F*	d.f.	*P*
Striped Shiner	Sangamon River	Body size	0.63	24.1	8, 113	<0.001
*Luxilus chrysocephalus*	(1901–2001)	Discharge mean	0.24	4.6	8, 113	<0.001
		Discharge CV	0.29	5.9	8, 113	<0.001
		Sex	0.11	1.6	8, 113	0.14
		Year	0.49	13.7	8, 113	<0.001
		Body size * discharge mean	0.16	2.7	8, 113	0.01
		Body size * discharge CV	0.15	2.4	8, 113	0.02
		Discharge mean * year	0.22	3.9	8, 113	<0.001
		Discharge CV * year	0.35	7.7	8, 113	<0.001
		Sex * year	0.27	5.4	8, 113	<0.001
Redfin Shiner	Little Vermillion River	Body size	0.18	2.3	7, 73	0.04
*Lythrurus umbratilis*	(1899–2002)	Discharge mean	0.14	1.7	7, 73	0.11
		Discharge CV	0.08	0.85	7, 73	0.55
		Sex	0.28	3.9	7, 73	<0.001
		Year	0.21	2.8	7, 73	0.01
		Body size * discharge mean	0.23	3.1	7, 73	<0.001
		Discharge mean * year	0.26	3.6	7, 73	<0.001
Emerald Shiner	Illinois River	Body size	0.35	4.3	8, 71	<0.001
*Notropis atherinoides*	(1897–2003)	Discharge mean	0.23	2.7	8, 71	0.02
		Discharge CV	0.69	19.5	8, 71	<0.001
		Sex	0.32	4.2	8, 71	<0.001
		Year	*	*	*	*
	Lake Michigan	Body size	0.51	14.8	6, 85	<0.001
	(1900–1999)	Sex	0.27	5.3	6, 85	<0.001
		Year	0.41	9.2	6, 85	<0.001
		Body size * sex	0.19	3.2	6, 85	<0.01
		Sex * year	0.17	2.9	6, 85	<0.01
Sand Shiner	Sangamon River	Body size	0.39	8.6	8, 108	<0.001
*Notropis stramineus*	(1901–2005)	Discharge mean	0.41	9.3	8, 108	< 0.001
		Discharge CV	0.42	9.7	8, 108	<0.001
		Sex	0.24	4.2	8, 108	<0.001
		Year	0.11	1.6	8, 108	0.14
	Mackinaw River	Body size	0.3	3.5	8, 64	<0.001
	(1901–2000)	Discharge mean	0.19	1.9	8, 64	0.05
		Discharge CV	0.08	0.69	8, 64	0.71
		Sex	0.11	0.95	8, 64	0.48
		Year	*	*	*	*
Bluntnose Minnow	Salt Creek	Body size	0.61	11.1	7, 49	<0.001
*Pimephales notatus*	(1900–2003)	Discharge mean	0.24	2.2	7, 49	0.05
		Discharge CV	0.06	0.4	7, 49	0.87
		Sex	0.42	5.1	7, 49	<0.001
		Year	0.16	1.3	7, 49	0.26
		Body size * sex	0.38	4.4	7, 49	<0.001
	Lake Michigan	Body size	0.64	17.5	7, 68	<0.001
	(1900–1998)	Sex	0.31	4.4	7, 68	<0.001
		Year	0.18	2.1	7, 68	0.05

See Figs. 3, 4, 5, 6, 7 for visualizations of shape changes

### Cyprinidae

The combined analysis at the family level included 908 individuals from 68 lots representing both lotic and lentic sites spanning 1897–2003. Total morphological variation was captured along 18 relative warp axes which explained 100 % of shape variation. However, shape was primarily driven by the first six axes which accounted for 80 % of this variation. The primary morphological gradient extracted from the family level MANCOVA model (aside from the species grouping variable) was body size, which explained 45 % of the total morphological variation. In addition to body size, sex (11 %) and year (8 %) were also recovered as important contributors to morphological variation. In lotic populations, both discharge mean (11 %) and variation (8 %) were recovered across all taxa. Testing for differences in trajectories of these relationships between species through the species interaction term uncovered significant variation in all cases indicating that there are species level differences in body shape responses to size, sex, year, and hydrology (Table 3). These differences in overall trajectory or slope between species renders an overall visualization less useful than discerning species specific trends as presented in subsequent results with species level visualizations. However, relative to overall morphological variation, disparity analysis indicated significantly different levels of variation across species (P < 0.05) and that the highest amount of morphological variation was found in Striped Shiner followed by Redfin Shiner, Emerald Shiner, Bluntnose Minnow, and Sand Shiner, respectively. Interesting, both species with lake populations, Emerald Shiner and Bluntnose Minnow, exhibited appreciably more variation in the lentic populations compared with their lotic counterparts while the only species with multiple lotic samples (Sand Shiner) were not significantly different from one another.

### Striped Shiner

Striped Shiner collections were from a single lotic site, the Sangamon River, and included 131 individuals from nine lots spanning 1901–2001. Total morphological variation was captured along 18 relative warp axes which explained 100 % of shape variation. This variation was primarily driven by the first 8 relative warp axes which accounted for 87 % of the shape variation. The primary morphological gradient extracted from the Sangamon River MANCOVA model was body size, which explained 63 % of the total morphological variation. Smaller individuals tended to have narrower body, caudal peduncle areas, and fin bases compared to larger individuals. Secondarily, year (49 %) was identified as an important predictor of morphology as individuals from more recent collections tended to exhibit more fusiform morphologies than the deeper bodied individuals from past collections. Hydrological analyses of the Sangamon River site provide a potential causal mechanism that undergirds this year variable. IHA analyses showed significant alterations to 9 (of 32) different types of hydrological variables between 1910 and 2005 (Appendix). Regression slopes for IHA variables with time indicated that the largest hydrologic alteration types occurred in magnitude and duration of annual extremes. Although it was not possible to explicitly incorporate IHA variables into the morphological model, a more direct test of hydrology, variation in annual discharge (29 %) and mean annual discharge (24 %), also supported a relationship between shape and hydrological variation. Striped Shiner morphologies with downturned heads, shorter and more robust caudal peduncle areas, and generally deeper bodies coincided with higher annual discharge and higher variation in annual discharge. A significant interaction between both year and annual discharge mean, as well as variation in discharge, indicated that something outside of these hydrological parameters is also likely influencing morphological variation independent of time. This variation could be attributed to some other hydrological attribute, such as one measured by the IHA, or reflect an unmeasured habitat variable that has also changed with time. Additionally, interactions recovered between body size and hydrology metrics (daily mean and variation in daily mean) indicated that while there was an overall effect of hydrology on morphology, this relationship was not consistent across all sizes of individuals. Lastly, interactions between sex and year indicated that males and females responded to the time variable differently, despite the lack of overall significance between parent terms. No interaction between sex and hydrology was recovered. See Table 4 and Fig. 3 for model configuration and deformation grids depicting shape change.

### Redfin Shiner

Redfin Shiner collections were from a single lotic site, the Little Vermillion River, and included 104 individuals from nine lots spanning 1899–2002. Total morphological variation was captured along 18 relative warp axes which explained 100 % of shape variation. This variation was primarily driven by the first seven relative warp axes which accounted for 81 % of the shape variation. The primary morphological gradient extracted from the MANCOVA model was sex, which explained 28 % of the total morphological variation. Females were found to exhibit deeper abdomens than males, however, this was not found to relate with any interactions with body size, hydrology, or year indicating consistent differences among body sizes, hydrology variables, sex differences, and year. Secondarily, year (28 %) and body size (18 %) were both significant main factors in morphological variation. More recent collections tended to exhibit comparatively smaller head shapes, a more arched dorsal surface, increasingly posterior pelvic and dorsal fin placements, and deeper caudal peduncles than older collections. Related to time, IHA analyses of the Little Vermillion River site showed significant hydrologic alterations in 19 (of 32) different types of IHA variables between 1915 and 2002 (Appendix). Regression slopes for IHA variables with time indicated that the largest alterations occurred in the magnitude and duration of annual extremes. Relative to body size, larger fish were found to be deeper bodied with shorter head areas compared with smaller bodied individuals. Although neither mean daily discharge or variation in daily discharge were significant, the model did recover interaction between mean daily discharge and time as well as body size, related to 26 % and 23 % of the variation, respectively. See Table 4 and Fig. 4 for model configuration and deformation grids depicting shape change.

### Emerald Shiner

Emerald Shiner collections were from a single lotic site, the Illinois River, and one lentic site, Lake Michigan, and included 198 individuals from 15 lots spanning 1897–2003. Total morphological variation for Illinois River and Lake Michigan individuals was captured along 18 relative warp axes which encompassed 100 % of the variation in each analysis, but was primarily driven by 8 and 6 relative warp axes that accounted for 86 and 84 % of shape variation Illinois River and Lake Michigan populations, respectively.

The primary morphological gradients extracted from the Illinois River model were hydrological, including variation in annual discharge (69 %) and mean annual discharge (23 %). More fusiform individuals with downward turned and extended caudal peduncle areas tended to occur during periods of lower mean annual discharge and higher variation in this discharge. In addition to hydrology, shape of Illinois River individuals also correlated with variation in body size (35 %) and sex (32 %). Larger individuals tended to exhibit deeper bodies, more robust caudal peduncles, and narrower heads compared with smaller individuals. In addition, females also tended to exhibit deeper bodies with more robust caudal peduncles compared with males. Issues with multicollinearity (VIF > 2) necessitated the exclusion of year from the Illinois River model. No interaction terms were recovered in this model, indicating a lack of sex-specific allometry or hydrological covariates. Although time was excluded from the model due to issues with multicollinearity, hydrological analyses of the Illinois River site revealed significant hydrologic alterations to 23 (of 32) IHA variables between 1920 and 2003 (Appendix). Regression slopes for IHA variables with time indicated that the largest alterations occurred with magnitude and duration of annual extremes.

The primary morphological gradients extracted from the Lake Michigan model were body size (51 %), year (41 %), and sex (27 %). Similar to collections from the Illinois River, larger individuals and females tended to be deeper bodied with more upturned heads and comparatively robust caudal peduncle areas. Interestingly, a significant interaction between body size and sex (19 %), not recovered in the Illinois River model, was recovered in the Lake Michigan model, indicating a degree of sex-specific allometry present in Lake Michigan collections. Lastly, year was explained by more recent collections of increasingly fusiform shaped individuals with upturned and narrower head and caudal shapes compared with older collections. This relationship between body shape and time was compounded by an interaction with sex, indicating a difference in degree of response to time between males and females. See Table 4 and Fig. 5 for model configuration and deformation grids depicting shape change.

### Sand Shiner

Sand Shiner collections were from two lotic sites, the Sangamon River and Mackinaw River, and included 306 individuals from 20 lots spanning 1901–2005. Total morphological variation explained for Sangamon River and Mackinaw River individuals was captured along 18 relative warp axes that explained 100 % of the total variation in each population. This variation was primarily driven by eight axes in each case which accounted for 84 and 83 % of the total morphological variation among Sangamon and Mackinaw River individuals, respectively. Among the primary morphological gradients extracted from both the Sangamon River and Mackinaw River MANCOVA models was body size, which explained 39 and 30 % of the total morphological variation, respectively. Both sites exhibited similar allometric shape trends related to increased dorsal fin base, anterior placement of pectoral fin, as well as deepening of the body and head concurrent with increased body size. Sangamon River populations were further described by high levels of sex-specific (24 % variance explained) differences, primarily related to more distended abdomens in females compared with males. Lack of an interaction between body size and sex retained in the final model indicated homogeneity of slopes between males and females in the size range included in this analysis, despite differences in overall body shape. This high degree of sex difference in body shape was different from Mackinaw River populations where sex was related to only 11 % of variation and was not recovered as statistically significant in the final model. Hydrological variables provided explanation for Sangamon River and Mackinaw River morphologies as higher levels of mean discharge for both the Sangamon (41 %) and Mackinaw (19 %) Rivers were positively related to deeper bodied and more robust individuals. Higher levels of variation in mean daily discharge tended to relate (42 %) to increasingly fusiform shapes with higher arched dorsal surfaces in Sangamon River populations only. Lastly, time was not recovered as a significant predictor of morphology in either model as issues with multicollinearity (VIF > 2) warranted exclusion of year from the Mackinaw River model and nonsignificant model terms precluded interpretation in the Sangamon River model. However, hydrological analyses of the Mackinaw River site elicited significant hydrologic alterations to 1 (32) type of IHA variable between 1922 and 2000 (Appendix). Regression slopes for IHA variables with time indicated a reduction in high pulse numbers in more recent Mackinaw River years. Similarly, hydrological analyses of the Sangamon River showed significant alterations to 9 (of 32) of the measured IHA type variables between 1910 and 2005 (Appendix). Regression slopes for IHA variables with time indicated that the largest alterations in the Sangamon River occurred in magnitude and duration of annual extremes. No relationships were recovered between sex and hydrology, sex and body size, or sex and time at either site, indicating similar slopes. See Table 4 and Fig. 6 for model configuration and deformation grids depicting shape change.

### Bluntnose Minnow

Bluntnose Minnow collections were from one lotic site, Salt Creek, and one lentic site, Lake Michigan, and included 169 individuals from 15 lots spanning 1900–2003. Total morphological variation explained for Salt Creek and Lake Michigan individuals was captured along 18 relative warp axes that explained 100 % of the total variation in each population. This variation was primarily driven by seven axes in each case which accounted for 83 and 87 % of the total morphological variation among Salt Creek and Lake Michigan individuals, respectively. The primary morphological gradient extracted from both Salt Creek and Lake Michigan MANCOVA models was body size, which explained 61 and 64 % of the total morphological variation, respectively. Both sites exhibited similar shape trends with increasing body size, including an increasingly downward facing snout, deeper body, and more robust caudal region. In addition to body size, both sites exhibited significant variation attributable to sex (42 and 31 %, respectively). Females from both sites differed from males by deeper abdomens and less overall arching of the dorsal surface. Collections from Salt Creek also demonstrated strong signal (38 %) from the body size and sex interaction term, indicating a higher degree of sex-specific allometric slope differences compared with Lake Michigan individuals. Relative to time, collections from Lake Michigan (18 %) indicated that individuals from collections in recent years had longer and narrower caudal regions, more terminal snouts (compared with superior snouts in past years), more fusiform overall body shapes, and a reduction in dorsal fin base length than past years. Time did not have a significant effect on morphology of individuals from Salt Creek. However, hydrological analyses of Salt Creek site during this time identified significant alterations in 6 (of 32) types of IHA variables between 1942 and 2005 (Appendix). Regression slopes for IHA variables with time indicated that the largest hydrologic alterations occurred in magnitude and duration of annual extreme flow events. Salt Creek hydrology variables supported a relationship between morphology and daily discharge mean (24 %) as higher values coincided with increasingly fusiform individuals compared with more robust individuals typical of lower daily discharge mean values. Salt Creek Bluntnose Minnow morphological variation over these years did not demonstrate a significant effect from time. Similarly, no interaction terms were recovered between sex and hydrology, body size and hydrology, or sex and year. See Table 4 and Fig. 7 for model configuration and deformation grids depicting shape change.

## Discussion

Morphological models identified significant shape relationships with body size, sex, hydrology, time, and a series of interactions in this exploratory study of fish shape variation over the past 100 years. Several trends emerged that indicated similarities and differences in the relative contributions of each variable. However, no universal pattern or formula described Cyprinidae shape over the past century consistently across species. Overall, body size trends tended to be similar among taxa as larger individuals tended to be deeper bodied, exhibit narrower head shapes, show arching of the dorsal surface, and have compressed caudal peduncle regions relative to smaller individuals. In addition, sex-specific differences among species were present in the species measured and were generally driven by females which exhibited deeper or distended abdominal regions compared with males. In lotic sites only, responses to hydrological variables such as mean annual discharge or variation in discharge were present in most species but directionality of morphological responses was not consistent among species as some tended to be more fusiform with higher discharge or variation in discharge, while others demonstrated increased robustness. Lastly, time was recovered in almost every model as an important contributor to morphological variation, however directionality also varied between species. This variability associated with time was not surprising, as the time variable is without a true mechanism measured over this century time span, and may reflect habitat variation at local and/or regional scales. Interactions among variables were far less consistent but did provide important controls for detecting differences in slopes between independent variables [42]. At the family level, the species level interaction term uncovered differences in slopes by species while at the intraspecific level interaction terms uncovered variation in allometry, sexual dimorphism, responses to flow, and time.

### Body size and allometry

Morphological variation along body size gradients was expected as resource utilization in fishes typically changes during development into adulthood consistent with ontogenic relationships to niche [12, 13]. These aspects of body size and morphological relationships are frequently linked to prey selection, competition, microhabitat choice, and predator–prey dynamics [8, 51]. Results of this study supported a general change in morphology across species consistent with body size; however, directly linking this variation to local environments was not possible. An exception to this was information gleaned from the interaction terms with body size, as several species showed relationships between size and sex as well as size and hydrology, suggesting that the allometric scaling relationship is contingent on a variety of factors that may affect survival. Interestingly, these interactions were not consistent across taxa or sites. Moreover, the effect of body size was not consistent in strength of relationships across taxa. Consequently, this might suggest different levels of selection for the body shape and body size relationship across Cyprinidae.

### Sexual dimorphism

Morphological variation by sex was predicted as an effect of functional selection [52, 53] related to fecundity constraints due to ovary size relative to testes [54–56]. However, sexual shape dimorphism is not ubiquitous in Cyprinidae taxa [57]. Our results support this mixed result as sex-specific shape differences were not consistent across taxa or sites as evidenced by varying effects of sex between species and sites, as well as results failing to meet initial expectations of heterogeneity of body size slopes with sex. We suggest that this could be a result of either a real signal (or lack thereof) of sex-specific shape differences that are taxon-specific within Cyprinidae, or an artifact of the body size ranges available and used in this study. Interestingly, shape variation between males and females also seemed to fluctuate among sites, particularly for comparison of the lake to rivers, where lotic samples reflected a higher degree of sexual shape dimorphism in both species tested (Bluntnose Minnow and Emerald Shiner with lotic and lentic sites) than either of their lentic system counterparts. In addition, there were no interactions between sex and hydrology, where one might expect male morphologies to show a stronger response to flow than females given reproductive constraints on body shape [57]. Our results did not support our a priori hypotheses [52, 53] regarding constraints on female morphology with selection for increased fecundity, as both male and female fishes responded similarly to varying hydrology. It seems plausible that a factor beyond often cited fecundity constraints may be responsible for the varying signal. For example, subtle differences in shape between sexes may translate to variation in niche space (e.g. diet, resource utilization, etc.) and potential release of competition. More research in the field and lab is needed into the variability within and between species and between sexes that explore potential hypotheses relating to niche, hydrology, fecundity, and morphological variability.

### Hydrological influence

Changes in morphology with hydrology indicate the presence of environmental influences on morphology. And while hydrology is by no means the only potential environmental corollary of morphology, it represents a strong potential environmental mechanism to explain changes over time. This variation in morphology could be rooted in selective variation or occur as a result of phenotypic plasticity, however, these two potential mechanisms are not necessarily mutually exclusive. It is unlikely that field data without the addition of any population genetic component could be used to distinguish between the two. However, phenotypic plasticity likely contributes to the trends elicited in this study as several of the fishes exhibited exceptionally strong relationships with both mean annual discharge and with variation of discharge. Unfortunately, the relative contribution of plasticity in response to local environmental conditions or population genetic divergence to morphological variation is unknown for most taxa [11]. Determining whether plasticity, directional selection towards a specific morphology, or some combination thereof results in morphological covariation with the environment can further distinguish potential adaptations. However, this requires genetic information that is difficult to obtain following formalin fixation and long term ethanol museum storage [58].

This study complements contemporary hydrology and morphology studies, which typically only address hypotheses involving current environmental measurements and current fish samples by adding a long term component. The predominant trend among short term intraspecific morphology—hydrology studies is that individuals with deeper-bodied morphologies tend to occur in lower flow velocity reaches of lotic or in lentic ecosystems, whereas individuals with more fusiform morphologies tend to occur in high flow conditions [14–16]. We found that not all Cyprinidae taxa exhibited a significant signal with hydrology variation. Moreover, not all species demonstrated the same response to high levels of mean annual flow or variation of annual flows. High flow velocities are expected to select for morphology that is a fusiform shape, and the opposite with low flows because of the resistance—performance relationship resulting from hydrological drag in high vs low velocity waters that can accompany flow volumes [59]. One caveat that may explain the differences identified here is that high discharge does not necessarily translate to velocity, and in fact may have an opposite effect, as higher volumes can overflow into floodplains, resulting in reaches with relatively low velocity. Furthermore, although not a direct objective of this study, visualization of consensus shapes between lentic and lotic sites suggested that individuals from lentic sites tended to be deeper-bodied than lotic individuals. However, future studies should directly quantify this with additional Cyprinidae species to test whether the pattern emerges across all species or is taxon specific since this study was based more on site level replicates through time and less on spatial replicates [26].

### Long term habitat change

In this study, while hydrology served as a direct mechanism of morphological change, time was used an indirect metric of change and can function as a notice of some unmeasured abiotic or biotic alteration. In several instances, time was excluded because it was essentially synonymous with variation in included hydrology variables. In these instances, hydrology is likely the primary determinant of environmentally driven shape change. However, in other instances where time was not related to mean annual discharge or discharge variation, it provides a unique perspective in the MANCOVA models independent of the hydrological factors directly assessed. However, hydrological variation can be assessed using a variety of metrics or approaches and the IHA analyses elicited several other hydrological parameters that also fluctuated with time. Other variables, in addition to hydrological variation, that would improve understanding of Cyprinidae morphology include disentangling the relative contributions from microhabitat, individual movement and dispersal potential, community structure, and phylogenetic history [15, 60–66]. Incorporation of these additional details into Cyprinidae morphology studies may provide a better understanding of morphological evolution—and by extension improve understanding of large scale ecosystem, community, and conservation issues.

### Systematic, ecosystem, and community implications

Temporal variation in morphology can result from immigration, selection, or other causes [67]. Therefore the presence of temporal morphological variation can prevent or disrupt long term ecological conclusions based off of a single sample from a single site. This study utilized multiple samples from numerous sites for several species and could provide a useful template or model for understanding long term changes in the environment and resulting morphologies of fishes. The variation in morphology documented herein can contribute to our understanding of variation in large scale ecosystem processes in light of ecomorphology [7, 68–70]. Ecosystem attributes such as primary production, nutrient cycling, and stability are linked to functional richness and diversity [71]. Therefore, we may better understand how these changes can influence large scale processes by providing clear mechanisms to what drives form. For example, in a recent Cyprinidae study, Wanink and Witte [72] demonstrated morphological differentiation that coincided with a pelagic to benthic niche shift as a result of an ecological disturbance. Similarly, Eklöv and Svanbäck [9] identified a predator- and habitat-induced shift in morphology that coincided with resource specialization. However, despite few studies that documented linkages between morphological variation and ecosystem attributes, more direct comparisons or experiments are necessary. This study describes morphological variation that coincides with several metrics that relate to organismal function. As morphology changed with time as documented in the model one can postulate potential shifts in the ecosystem pertaining to energy, habitat, or other members of the community. However, studies that directly address the hypothesis of ecosystem covariation with intraspecific morphological variation may be confounded by multiple interspecific population responses contributing to an ecosystem process. In addition, hydrological alterations directly influence ecosystem processes and are present in the majority of lotic and lentic systems worldwide [25]. Thus, the ability to manipulate a single population within an ecosystem and measure an indirect effect of morphological change on ecosystem properties may be possible only in laboratories or mesocosm experiments.

Morphological variation may also impact or influence community structure. Gatz [5] described resource utilization within an assemblage of freshwater stream fishes on the basis of their associated morphologies. He discerned similar morphological patterns among disparate assemblages indicating non-random distribution of resource niche space. Gatz’s [5] conclusion of non-random morphological assembly was supported by Winston [6] for Cyprinid shape, where a lack of morphological overlap was attributed to interspecific competition. This non-random distribution in morphological space within an assemblage could potentially result in large overall impacts following a shift in one or more taxa. Environmental alterations that induce a change in morphology and thus functional roles could instigate increased or relaxed competition, directly or indirectly.

## Conclusions

Discerning linkages between morphology and hydrology has conservation applications for understanding the capacity of species to respond to changing ecosystems. The results presented herein provide a template for species that have persisted in a variety of sites over the past century. Given that these sites are disturbed and altered (as evidenced by the IHA analyses), future work should attempt to repeat this study with different taxa from less altered sites to test whether these species are successful solely because they are plastic in their morphologies. Morphological variation may also aid in understanding the invasion potential for ecosystems [72] through competition overlap and resilience. One prediction of the ecosystem resilience model is that competition among taxa of similar shape [6] can prevent invasive taxa from becoming established [73]. Linking variation in morphology via disparity indices with assemblage shifts could provide one avenue to address these sorts of questions by establishing a metric based on morphology that could be used to look at ecosystem level resilience. Morphological variation within communities is linked to the zoogeographical history of a region [32] and can be applied at a taxon specific level to better understand historic and current environmental conditions and biogeographical patterns. Understanding individual response mechanisms may increase understanding of selective pressures that influenced the evolutionary history of the Cyprinidae.

## Appendix

See Tables 5, 6.

**Table 5:** Lot numbers for preserved specimens from the Illinois Natural History Survey (INHS)

Species	INHS	River	Year	Illinois county
*Luxilus chrysocephalus*	8513	Sangamon River	1965	Piatt
*Luxilus chrysocephalus*	16093	Sangamon River	1965	Macon
*Luxilus chrysocephalus*	21385	Sangamon River	1968	McLean
*Luxilus chrysocephalus*	43181	Sangamon River	1989	Champaign
*Luxilus chrysocephalus*	84335	Sangamon River	1901	Champaign
*Luxilus chrysocephalus*	84370	Sangamon River	1901	Champaign
*Luxilus chrysocephalus*	84385	Sangamon River	1901	Champaign
*Luxilus chrysocephalus*	91871	Sangamon River	2001	Champaign
*Luxilus chrysocephalus*	96038	Sangamon River	1988	Champaign
*Luxilus chrysocephalus*	96063	Sangamon River	1987	Champaign
*Luxilus chrysocephalus*	98050	Sangamon River	1988	Champaign
*Lythrurus umbratilis*	11950	Little Vermilion	1962	Vermilion
*Lythrurus umbratilis*	32769	Little Vermilion	1994	Vermilion
*Lythrurus umbratilis*	37727	Little Vermilion	1996	Vermilion
*Lythrurus umbratilis*	41540	Little Vermilion	1997	Vermilion
*Lythrurus umbratilis*	42177	Little Vermilion	1997	Vermilion
*Lythrurus umbratilis*	43139	Little Vermilion	1997	Vermilion
*Lythrurus umbratilis*	65032	Little Vermilion	1989	Vermilion
*Lythrurus umbratilis*	86476	Little Vermilion	1899	Vermilion
*Lythrurus umbratilis*	94588	Little Vermilion	2002	Vermilion
*Notropis atherinoides*	632	Lake Michigan	1964	Cook
*Notropis atherinoides*	25287	Illinois River	1957	Grundy
*Notropis atherinoides*	25460	Illinois River	1962	LaSalle
*Notropis atherinoides*	25480	Illinois River	1963	LaSalle
*Notropis atherinoides*	26591	Lake Michigan	1975	Cook
*Notropis atherinoides*	27303	Lake Michigan	1979	Lake
*Notropis atherinoides*	49876	Illinois River	1997	Grundy
*Notropis atherinoides*	51058	Illinois River	1902	LaSalle
*Notropis atherinoides*	51173	Lake Michigan	1998	Cook
*Notropis atherinoides*	52188	Illinois River	1999	LaSalle
*Notropis atherinoides*	53920	Lake Michigan	1999	Cook
*Notropis atherinoides*	84565	Lake Michigan	1900	Cook
*Notropis atherinoides*	85583	Illinois River	1897	Mason
*Notropis atherinoides*	98018	Illinois River	2003	LaSalle
*Notropis stramineus*	8022	Sangamon River	1959	Champaign
*Notropis stramineus*	10902	Mackinaw River	1958	Woodford
*Notropis stramineus*	14879	Mackinaw River	1963	Tazewell
*Notropis stramineus*	15063	Mackinaw River	1966	Tazewell
*Notropis stramineus*	16134	Sangamon River	1968	Macon
*Notropis stramineus*	21240	Sangamon River	1965	McLean
*Notropis stramineus*	33080	Mackinaw River	1994	Woodford
*Notropis stramineus*	40803	Mackinaw River	1995	Tazewell
*Notropis stramineus*	41475	Mackinaw River	1997	Tazewell
*Notropis stramineus*	42050	Mackinaw River	1996	Tazewell
*Notropis stramineus*	42188	Sangamon River	1997	Champaign
*Notropis stramineus*	52941	Mackinaw River	1999	Woodford
*Notropis stramineus*	57216	Sangamon River	2000	Macon
*Notropis stramineus*	84387	Sangamon River	1901	Champaign
*Notropis stramineus*	85832	Mackinaw River	1901	McLean
*Notropis stramineus*	93187	Mackinaw River	2000	McLean
*Notropis stramineus*	93223	Mackinaw River	2000	McLean
*Notropis stramineus*	96042	Sangamon River	1988	Champaign
*Notropis stramineus*	99815	Sangamon River	2003	Champaign
*Notropis stramineus*	101741	Sangamon River	2005	Macon
*Pimephales notatus*	617	Lake Michigan	1964	Cook
*Pimephales notatus*	13714	Salt Creek	1963	DeWitt
*Pimephales notatus*	13731	Salt Creek	1967	DeWitt
*Pimephales notatus*	18681	Salt Creek	1971	Logan
*Pimephales notatus*	42450	Salt Creek	1997	DeWitt
*Pimephales notatus*	42834	Salt Creek	1997	Logan
*Pimephales notatus*	56877	Lake Michigan	1980	Cook
*Pimephales notatus*	84567	Lake Michigan	1900	Cook
*Pimephales notatus*	85438	Salt Creek	1900	Logan
*Pimephales notatus*	85460	Salt Creek	1901	Logan
*Pimephales notatus*	89383	Salt Creek	2000	Mason
*Pimephales notatus*	96955	Lake Michigan	1999	Cook
*Pimephales notatus*	96960	Lake Michigan	1998	Cook
*Pimephales notatus*	97146	Lake Michigan	1987	Cook
*Pimephales notatus*	97588	Salt Creek	2003	Mason

**Table 6:** Results from IHA analyses with time

Variable	IHA category	Illinois River		Mackinaw River		Salt Creek		Sangamon River		Vermillion River	
		Slope	R^2^	Slope	R^2^	Slope	R^2^	Slope	R^2^	Slope	R^2^
High pulse count	Frequency and duration of pulses	*0.03*	0.05	*−0.07*	0.18	−0.02	0.02	*0.03*	0.07	*0.05*	0.1
High pulse duration		−0.02	0.01	0.15	0.06	*0.14*	0.09	0.07	0.04	−0.01	0.01
Low pulse count		*0.16*	0.19	0	0	0.01	0	0	0	0.01	0.01
Low pulse duration		*0.04*	0.07	−0.05	0.01	−0.1	0.01	*−0.62*	0.08	*−0.19*	0.09
1-day maximum	Magnitude and duration of annual extremes	*295.2*	0.15	−15.08	0	21.41	0	129.6	0.04	*81.05*	0.06
1-day minimum		*−61.55*	0.51	−0.1	0.02	*0.84*	0.12	*1.74*	0.07	*0.44*	0.18
30-day maximum		*58.76*	0.05	2.91	0	21.19	0.03	*58.36*	0.06	*17.57*	0.07
30-day minimum		*−65.06*	0.49	−0.4	0.05	*1.31*	0.1	0.48	0	*0.75*	0.13
3-day maximum		*241.7*	0.14	−1.38	0	21.84	0	122.8	0.04	*65.77*	0.07
3-day minimum		*−63.79*	0.55	−0.12	0.02	0.80	0.11	*1.62*	0.06	*0.46*	0.18
7-day maximum		*168.40*	0.12	−0.08	0	18.83	0	*101.8*	0.05	*37.48*	0.06
7-day minimum		*−64.05*	0.55	−0.12	0.02	*0.99*	0.12	1.51	0.04	*0.48*	0.17
90-day maximum		6.55	0.00	1.4	0	13.7	0.04	*33.14*	0.05	*10.19*	0.08
90-day minimum		*−51.73*	0.29	−0.09	0	1.73	0.01	4.17	0.02	*3.02*	0.06
Baseflow index		0	0.56	0	0.01	0	0.03	0	0.02	*0*	0.06
April	Magnitude of monthly conditions	−38.09	0.02	−4.48	0.02	2.82	0	−1.15	0	5.77	0.03
August		*−41.95*	0.17	1.29	0.03	6.62	0.03	−9.85	0.01	2.21	0.02
December		*−42.04*	0.06	−1.51	0.01	9.13	0.03	*31.28*	0.08	*6.32*	0.06
February		*−39.67*	0.05	−3.34	0.02	3.78	0	14.22	0.01	3.02	0.01
January		*−61.71*	0.11	−1.32	0	0.36	0	4.07	0	−5.45	0.02
July		−11.87	0.01	2.99	0.03	4.49	0.01	−1.19	0	3.07	0.02
June		3.09	0.00	4.72	0.07	10.42	0.03	13.86	0.01	4.24	0.03
March		−18.53	0.01	1.18	0	19.43	0.06	27.55	0.04	*8.99*	0.07
May		−25.76	0.02	7.83	0.04	17.66	0.04	29.63	0.03	4.31	0.01
November		*−51.93*	0.10	−0.78	0	2.28	0	10.67	0.02	*6.26*	0.05
October		*−52.70*	0.13	−1.31	0.01	4.14	0.01	−0.31	0	*3.48*	0.05
September		*−38.86*	0.07	0.41	0	5.15	0.04	−12.2	0.02	1.49	0.01
Fall rate	Rate and frequency of water condition changes	*−3.67*	0.18	0.07	0	−0.11	0	−0.32	0.01	*−0.37*	0.08
Number of reversals		*0.47*	0.11	0.12	0.03	0.12	0.03	*0.52*	0.41	*0.36*	0.17
Rise rate		*1.98*	0.05	−0.54	0.02	−0.13	0	−0.15	0	0.47	0.02
Date of maximum	Timing of annual extreme conditions	0.47	0.02	0.4	0.02	*1.07*	0.07	−0.06	0	0.19	0
Date of minimum		*0.92*	0.05	−0.02	0	−0.1	0	0.43	0.02	−0.08	0

See Tables 1 and 2 for site descriptions. Significant (P < 0.05) slope values are in italic

## Abbreviations

INHS: Illinois Natural History Survey
USGS: United States Geological Survey
IHA: indicators of hydrologic alteration
GPA: generalized procrustes analyses
RWA: relative warp analysis
MANCOVA: multivariate analysis of covariance
VIF: variance inflation factor
